# Strategies for screening young stock for antibodies – optimising numbers to test, cut-points, & predictive values for bovine viral diarrhoea virus

**DOI:** 10.1038/s41598-018-27870-8

**Published:** 2018-06-22

**Authors:** R. W. Humphry, A. Reeves, G. J. Gunn

**Affiliations:** Epidemiology Research Unit, (Inverness campus), Scotland’s Rural College (SRUC), Kings Buildings, West Mains Road, Edinburgh, EH9 3JG UK

## Abstract

The antibody seroprevalence of young stock can be a useful indicator of recent or current infection in a herd. We examine the factors that contribute to the assessment of herd exposure to disease, via spot testing for antibody, using bovine viral diarrhoea virus (BVDv) as an example. A statistical distribution of seroprevalences for BVDv in beef herds identified three groups of herds: low, intermediate and high within-herd BVDv antibody seroprevalence. We tested two assumptions –the intermediate seroprevalence group of herds is assumed to be negative for BVDv at the herd level and alternatively if this group is assumed to be positive. We found that: The herd-level sensitivity and specificity are sensitive to the assumption regarding the herds with intermediate seroprevalence. If an appropriate cut-point is chosen, reducing the sample size from ten to five does not produce a large drop in herd-level test performance. Increasing the cut-point may be valuable at the outset of an eradication programme. Increasing the sample size and decreasing the cut-point is advantageous towards the end of an eradication programme, to minimise the risk of positive herds being misclassified. The framework presented here illustrates how seroprevalence screening may be understood and assessed.

## Introduction

For most infectious agents, antibodies measured in animals by an ELISA test provide information about the recent or historic exposure of those animals to the infectious agent. The prevalence of animals with high antibody levels (*i*.*e*., antibody seroprevalence) may thus be used to identify herds that have been recently or currently are being exposed to the infectious agent. This principle has been employed in the screening of herds for infectious diseases, such as bovine viral diarrhoea virus (BVDv) in cattle.

BVDv is the infectious agent of a serious cattle disease of economic importance^1,2^. BVDv is amenable to eradication at a national level if suitable testing and management in response to testing are put into action^3,4^. It is possible to test directly for antigen to BVDv amongst individual animals but, if missed, an infectious animal can cause serious break down in status for the herd and result in further cycles of infection. An alternative method of identifying BVDv-infected herds involves the screening of animals for antibodies and the use of screening results as an indicator of virus circulating in the herd. For this primary screen based on antibodies it is common to screen young stock since their antibody levels are indicative of recent infection and it is the recent status of the herd which is typically of most interest^5^. If a herd is deemed to be BVDv-positive (*i*.*e*., infected) on the basis of the detection of antibody-positive animals, a subsequent and more extensive search for antigen-positive (and potentially infectious) animals within the herd typically will be conducted. A particular aim of such subsequent screening is the identification of persistently infected (“PI”) animals, which are epidemiologically important for the continued spread of disease^6,7^.

Using the presence of antibody-positive animals to identify infected herds is dependent upon several factors. Levels of within-herd antibody seroprevalence have been found to vary across populations of herds^5,8^. The variation in seroprevalence amongst herds that are truly BVDv-positive as well as among those that are truly BVDv-negative (i.e. not infected) will impact the results of a screening test. Among the other factors that will affect the performance of a screening test are the following:

- The sensitivity and specificity of the antibody test at the level of the individual animal;
- The number of animals to be tested or sampled; and
- The use of an appropriate cut-point or threshold for the number of test-positive animals in the sample at which the herd (or flock) is deemed positive.

Together, these factors contribute to the specificity and sensitivity of the screening test at the level of the whole herd.

Screening programmes for BVDv which utilise testing for antibodies in individual sera from young stock vary in the number of animals they require to be tested and in the cut-point, *i*.*e*., the number of seropositive animals which must be detected in the sample in order for the herd to be deemed positive. For example, the first mandatory testing stage of the Scottish eradication scheme^9^ required five or ten young animals to be tested per management group depending on the age of the young stock with an implicit cut-point of one positive animal albeit with scope for reinterpretation by the veterinarian in the case of low numbers of animals with low or inconclusive antibody levels^10^.

Thus the detection of even a single antibody seropositive animal in the sample was sufficient for the herd to be deemed positive for BVDv. In the eradication programme proposed for the Netherlands an initial screening of five young stock leads to additional testing if two or more tested animals are found to be antibody positive (Pers. Comm. Duijn, L. van 8/6/17).

**Figure 1:**
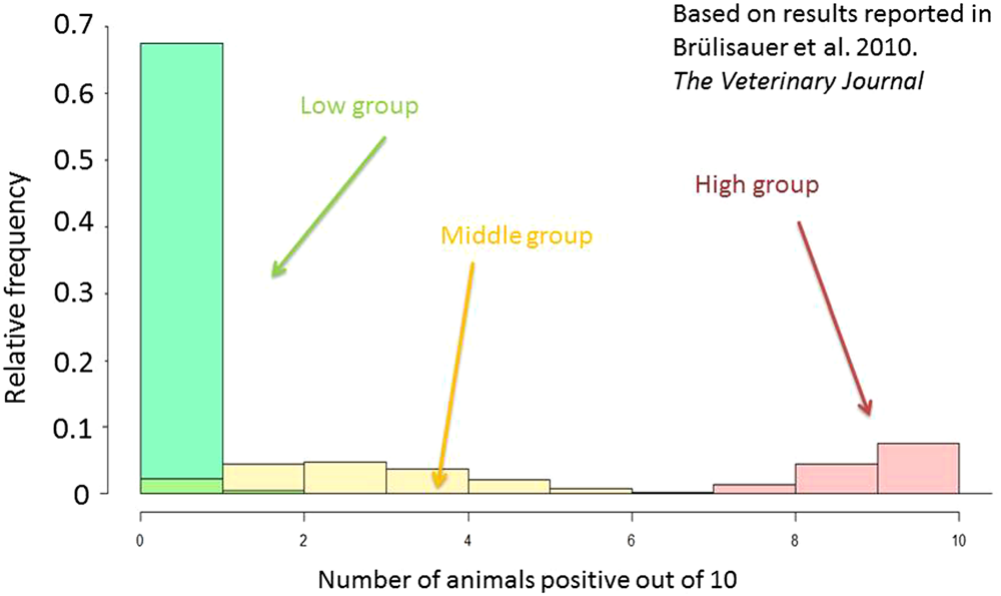
A graphical presentation of the three groups of herds identified and described on the basis of different levels of antibody seroprevalence in a study involving seroprevalence of ten young stock from Scottish beef suckler herds^8^.

As noted above, the within-herd BVDv antibody seroprevalence varies among herds. The statistical distribution of seroprevalence amongst young stock is generally U-shaped with a large proportion of herds with zero or close to zero seroprevalence and a large proportion with close to 100% seroprevalence^5^. However, there is also evidence of a middle group or class of herds with intermediate seroprevalence (Fig. 1)^8,11,12^. It is not clear whether herds in this group are truly BVDv-positive, BVDv-negative or a mix of both. How herds in this middle group are considered has implications for BVDv screening. In this paper we estimate the herd-level sensitivity and specificity of screening tests under various conditions, considering the range of within-herd BVDv antibody seroprevalence levels, different numbers of animals tested and different cut-points for the threshold above which a herd is deemed positive. Our objective is to provide quantitative evidence for those designing schemes of the herd-level test performance of young stock screening for BVDv. An additional objective is to provide policy implications that vary between the outset of an eradication scheme (when herd-level prevalence is high) and the latter stages of an eradication scheme (when false negatives are costly to the eradication scheme). We also consider the importance of the middle group and how it may affect the interpretation of the results of screening for antibodies in young stock.

## Results

**Figure 2:**
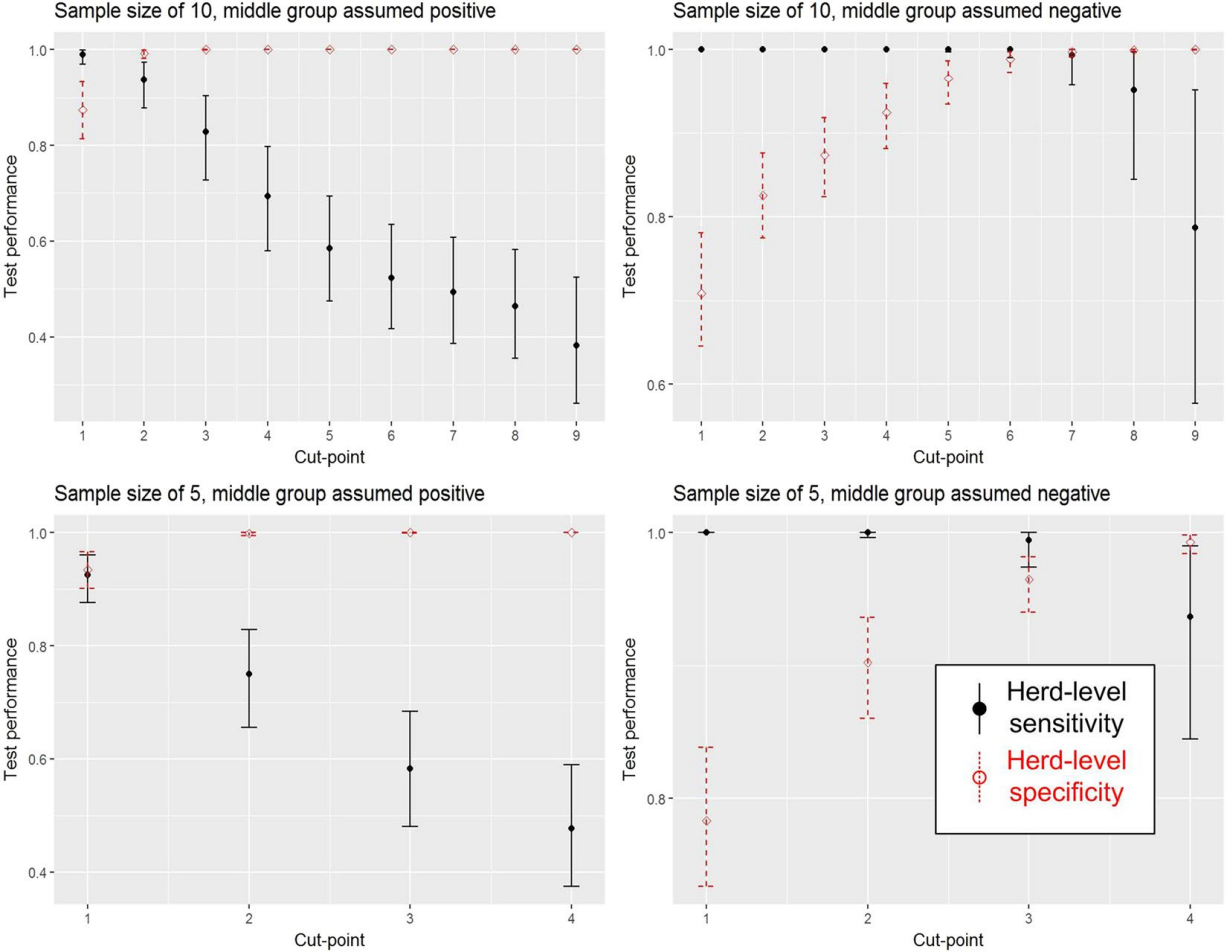
The herd-level test performance (sensitivity in black, specificity in red) where the group of herds with intermediate within-herd seroprevalence is treated as either positive or negative and using a sample size of either five or ten animals.

Results of test performance at the herd-level are presented for the following combination of scenarios (Fig. 2):

- Treatment of herds with intermediate levels of antibody seroprevalence as either BVDv negative or positive;
- Testing of five or ten animals; and
- Use of a cut-point or threshold (i.e. the number of positive animals at, or above which, the herd is declared positive) of different numbers of animals.

From results illustrated in Fig. 2, we note that the herd-level sensitivity drops and the herd-level specificity increases as the cut-point number of animals increases. It is also clear that the sensitivity is much higher and specificity lower if the group of herds with intermediate seroprevalence in the original distribution is considered negative rather than positive. The designation of the group of herds with intermediate levels of seroprevalence is very important in deciding the optimum cut-point. If this group is considered positive, then with a sample size of ten the optimum cut-point arguably is two positive animals. By contrast, if this group is considered negative, then the optimum cut-point is arguably six to achieve both high sensitivity and specificity. There is a similar pattern when considering a sample size of five animals. The optimum cut-point appears to be one positive in the case of the middle component being positive and three positives from a sample of five in the case of the middle component being negative.

Table 1 provides the herd-level performance estimates for a testing regime within a recommended control programme. Table 2 provides the overall probability of a false negative, the overall probability of a false positive, positive predictive value and negative predictive value under the eradication scheme in the Netherlands plus the consequences of reducing the cut-point r of seropositive animals from two to one.

**Table 1:** Median (& 95% percentile) herd-level sensitivity, specificity, false negative rate amongst positive herds and false positive rate amongst negative herds based on five animals and a cut-point of two seropositive animals (as written into the Netherlands’ eradication programme).

	If herds with intermediate within-herd seroprevalence are treated as positive	If herds with intermediate within-herd seroprevalence are treated as negative
Sensitivity	75% (66%, 83%)	100% (99.6%, 100%)
False negative rate amongst pos herds	25% (17%, 34%)	0% (0%, 0.4%)
Specificity	99.9% (99.4%, 100%)	90% (86%, 94%)
False positive rate amongst neg herds	0.10% (0%, 0.5%)	10% (6%, 14%)

**Table 2:** Overall probability of false negatives and positives based on a sample size of five young stock and the comparing the cut-point of two seropositive animals to one seropositive animal.

		If herds with intermediate within-herd seroprevalence are treated as positive		If herds with intermediate within-herd seroprevalence are treated as negative	
High herd-level prevalence (60% of herds)	Cut-point number of positive animals	1/5	2/5	1/5	2/5
	Overall Probability of False neg	4.5% (2.4%, 7.4%)	15% (10%, 21%)	0% (0%, 0%)	0% (0%, 0.2%%)
	Overall Probability of False pos	2.6% (1.3%, 3.9%)	0.06% (0%, 0.21%)	8.67% (6.46%, 10.66%)	3.92% (2.55%, 5.59%)
	Positive predictive value	95.5% (93.3%, 97.7%)	99.87% (99.5%, 100%)	87.4% (84.9%, 90.3%)	93.87% (91.47%, 95.92%)
	Negative predictive value	89.26% (83.51%, 93.98%)	72.67% (65.86%, 79.53%)	100.00% (100.00%, 100.00%)	100.00% (99.33%, 100.00%)
Low herd-level prevalence (**5% of herds)**	Overall Probability of False neg	0.04% (0.02%, 0.06%)	0.1% (0.09%, 0.17%)	0.00% (0%, 0%)	0.00% (0%, 0.002%)
	Overall Probability of False pos	6.5% (3.3%, 9.8%)	0.1% (0%, 0.52%)	21.57% (16.08%, 26.51%)	9.74% (6.34%, 13.90%)
	Positive predictive value	6.64% (4.47%, 12.29%)	71.25% (41.58%, 100.00%)	2.27% (1.85%, 3.02%)	4.88% (3.47%, 7.31%)
	Negative predictive value	99.96% (99.93%, 99.98%)	99.87% (99.83%, 99.91%)	99.96% (99.94%, 99.98%)	100.00% (100.00%, 100.00%)

The “false negative rate” is the probability that a truly positive herd tests negative (that is, the numerator is the number of truly positive herds which test negative, and the denominator is the number of truly positive herds)^13^. Possibly of greater interest to the policy maker is the probability that *any* herd randomly selected from a population of herds is truly positive *and* tests negative (the numerator is unchanged, but the denominator in this case is the total number of herds). Here we use the term “overall probability of a false negative” to distinguish this value from the false negative rate. The overall probability of a false negative is simply the true herd-level prevalence multiplied by the false negative rate.

**Figure 3:**
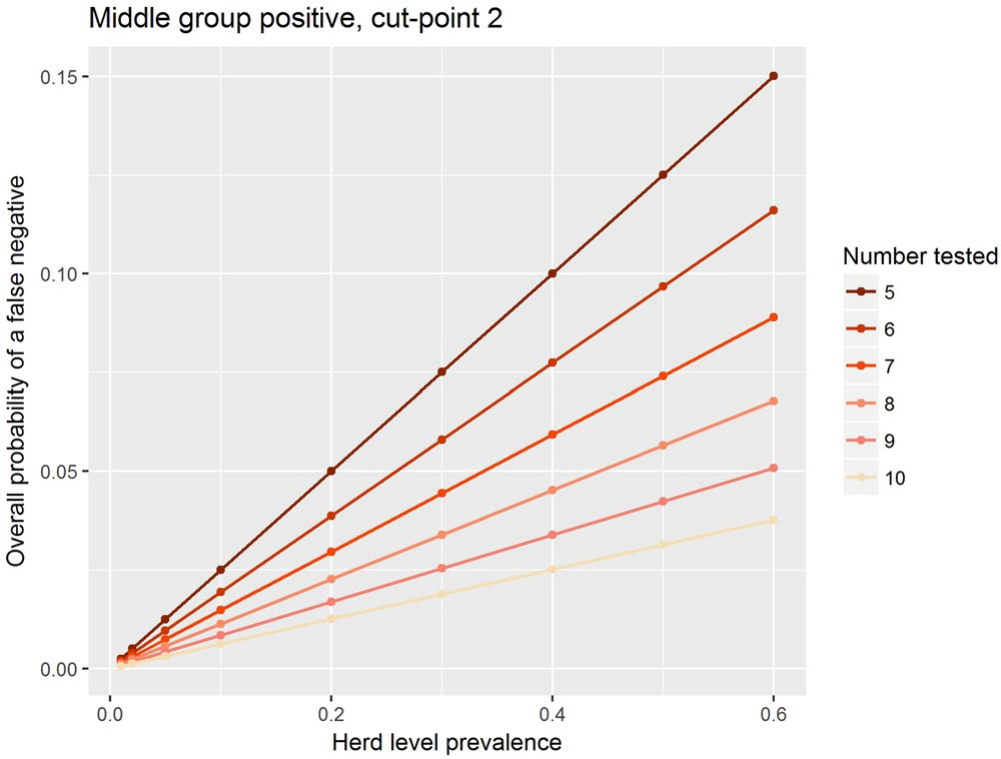
The median overall probability of false negatives with a cut-point of two positive animals and if herds with intermediate within-herd seroprevalence are treated as is positive.

**Figure 4:**
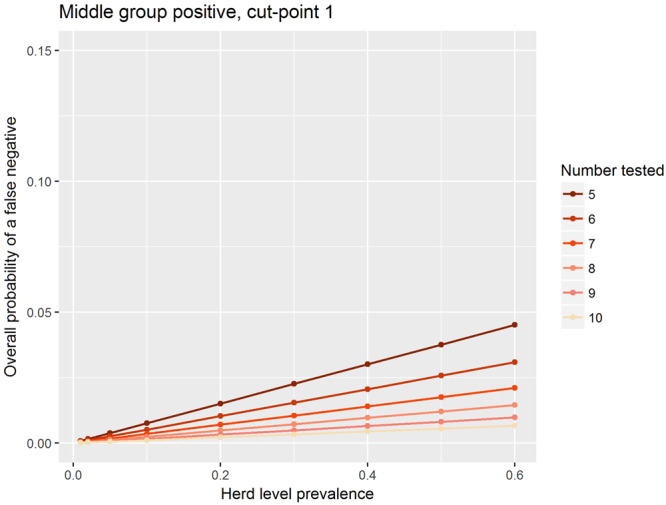
The median overall probability of false negatives with a cut-point of one positive animal and if the herds with intermediate within-herd seroprevalence are treated as positive.

**Figure 5:**
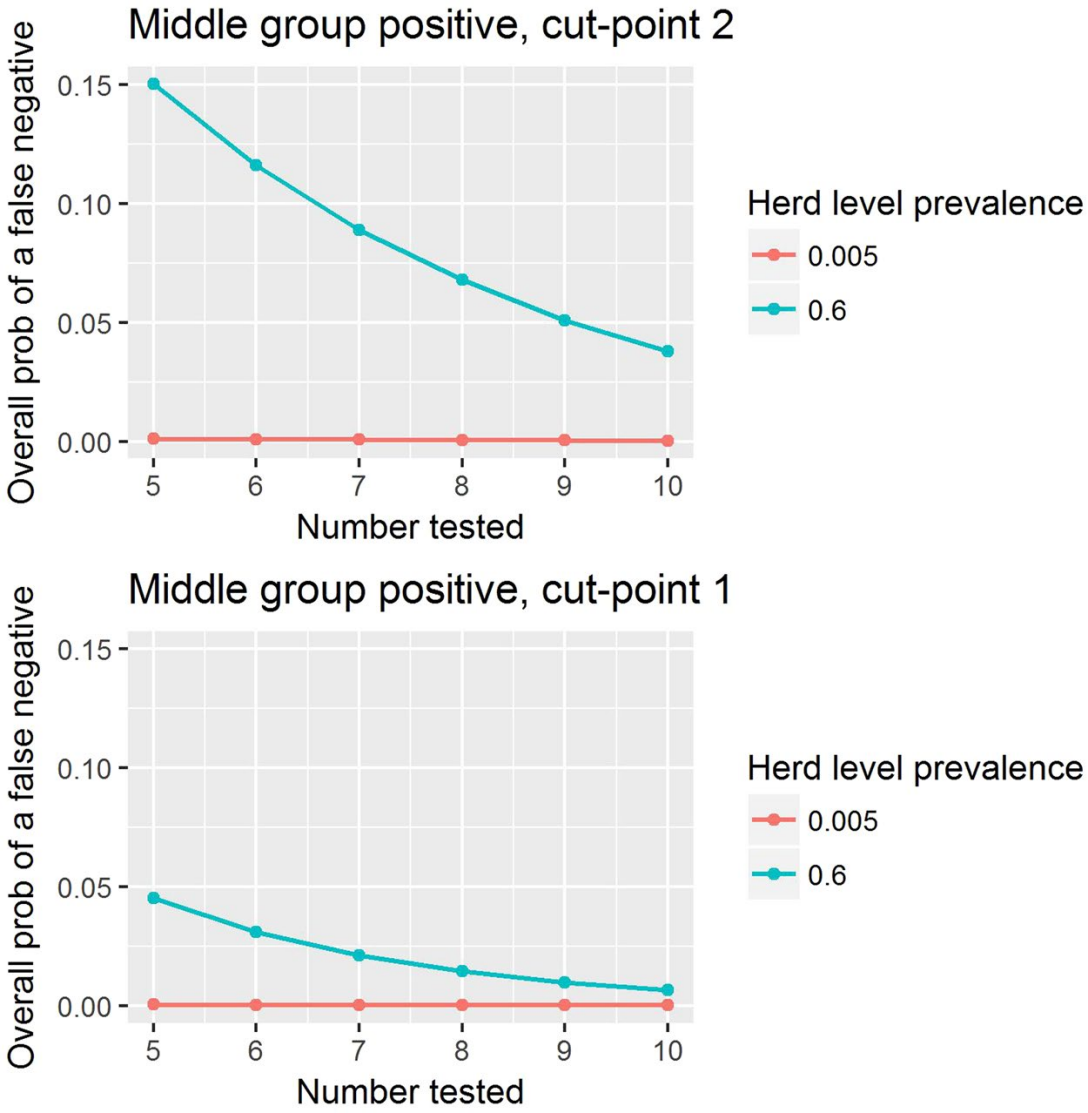
The median overall probability of false negatives as a function of the number of animals tested, two different prevalence levels (60% and 0.5%) and cut-points of one and two positive animals.

The overall probability of a false negative naturally increases linearly with herd-level prevalence (Figs 3 and 4). It is noticeable that by dropping the cut-point from two (Fig. 3) to one (Fig. 5, Table 2) the overall probability of a false negative is decreased substantially. For a given cut-point, increasing the number of animals tested lowers the overall probability of a false negative (Figs 2, 3 and 4).

We illustrate the consequences of our assumption for the status of the intermediate seroprevalence group for a scenario provided within the Dutch eradication scheme in which the number of animals tested is five and the cut-point is two (Table 1). This demonstrates how much the sensitivity and specificity of the test depends on this assumption with the sensitivity changing from 75% to 100% under the two assumptions.

In Table 2 we present the overall probability of false negatives and false positives. False negatives in particular are undesirable towards the latter stages of eradication when most herds and most animals are susceptible and therefore at risk of infection through the failure to identify a positive herd. We see that even though the overall probability of a false negative is lower when the prevalence is lower, the overall probability of a false negative can be reduced further by lowering the cut-point to one.

In Figs 3 and 4 we see the linear relationship between the herd level prevalence and the overall median probability of false negatives. We see that as the number of animals tested goes up for a fixed cut-point the overall probability of a false negative goes down. Finally we note that for any particular number of animals tested, if the cut point is reduced to one then the over all probability of a false negative decreases.

We do not present figures for the median overall probability of false negatives when herds with intermediate within-herd seroprevalence are treated as negative because in that situation the median rate of false negatives is zero for both a cut-point of one or two positive animals.

## Discussion

For most infectious agents, antibodies measured in animals by an ELISA test provide information about the recent or historic exposure of those animals to the infectious agent. The prevalence of animals with high antibody levels (i.e., seroprevalence) may thus be used to identify herds that have been or are being exposed to high levels of the infectious agent, provided that estimates of seroprevalence can be interpreted as a herd-level result. In this study we employ the example of BVDv to demonstrate how the herd-level sensitivity and specificity might be estimated on the basis of an interpretation of seroprevalence data collected from young stock in Scottish beef suckler herds.

### Relevant epidemiological attributes of BVDv

A natural gold standard for defining a herd as BVDv positive is evidence of circulating virus in the herd and therefore evidence of the presence of at least one persistently or transiently infected animal in the herd, which might be detected on the basis of screening animals for virus or for BVDv antigens. Such screening would require a great deal of testing (of every or nearly every animal in a herd), and the consequences of a false negative result could be substantial. Alternatively, however, the epidemiology of BVD virus is such that the presence of BVD virus in the herd is amenable to screening by testing for antibodies in animals of any age, and particularly in young stock. A study^14^ found that the within-herd antibody seroprevalence from herds in which an antigen-positive animal was identified was 87% whilst it was only 43% in herds in which no antigen-positive animal was found. Therefore the within-herd prevalence of antibody-positive animals may be effectively used as a proxy or indicator for the presence or absence of antigen-positive animals.

### Optimising the number of animals to test and the cut-point value

When a number of individual animals are tested for antibody in order to determine a binary (i.e., positive or negative) herd status, there are two important decisions to be made. The first is the number of animals to be tested and the second is the cut-point for the number of positive animals at or above which the herd is deemed positive. Commonly the number of animals to be tested in BVDv eradication programmes is 5–10 young stock^5,9,15–17^ although it has been suggested that as few as three young stock might need testing^17^ especially if the antibody test is done in tandem with antigen testing^18^. A BVDv eradication programme now being initiated in the Netherlands involves initial antibody spot-testing of five animals, with a cut-point for the number of antibody-positive animals of two. One clear benefit of choosing a cut-point of two (as opposed to one) is that it reduces the risk of false positives and thus reduces the risk of unnecessary and expensive secondary testing of the whole herd. Our work informs decisions regarding sample numbers and threshold values under different conditions.

The optimum threshold number, or cut-point, of antibody-positive animals for making a herd-level determination is dependent on the number of animals sampled. If ten animals are tested then the optimum cut-point appears to be greater than one (Fig. 2). This matches recommendations published elsewhere^5,19^. In our analysis the optimum appears to be two positive animals out of ten, if the intermediate seroprevalence group is considered positive and a cut-point of around seven positive animals if the intermediate seroprevalence group is considered to be negative (Fig. 2). Whilst it appears justified from an epidemiological point of view, using a cut-point of greater than one may be counter-intuitive to farmers and veterinary practitioners. To use a cut-point of greater than one is to lose some sensitivity of the test but to gain specificity i.e. to reduce the probability of false positives. As discussed below, the best balance between sensitivity and specificity is dependent upon the stage of a disease eradication programme.

### Performance of the individual level test

Our approach to estimating the *group*-level sensitivity and specificity is based on within-herd prevalence of antibody-positive animals, however it is worth noting that the *individual* tests are not perfect either. In the survey on which our simulations are based, the test used was Svanovir BVDV antibody ELISA^20^. This test is reported (by the manufacturer) to have a sensitivity and specificity of 100% and 98.2% respectively in comparison to a virus neutralisation test. In our simulations we have used the sensitivity and specificity from the statistical distributions reported in the study^8^ on which our simulations are based. The sensitivity (and 95% confidence interval) is 96.3% (91.9%, 99.8%) and the specificity is 98.8% (98.0%, 99.3%).

Our study is based on the categorisation of individual antibody scores into a binary positive or negative result by comparing the antibody scores with a cut-off. This is the conventional way of interpreting individual antibody results and is both convenient and easily interpreted. However, when we scale up to testing at the group level it is possible that the loss of information that is involved in categorising a continuous antibody score into a binary positive or negative is an important loss of information. For example, it is possible that having several calves with an antibody score just below the cut-off is a better predictor of a positive herd than having only one calf just above the cut-off. Current eradication schemes tend not to be set up to report individual antibody scores, and it is not known how frequently such a situation might occur. To take into account the individual antibody scores would require further investigation, ideally using data pertaining to individual antibody scores from sampled calves from herds with and without PI animals. The interpretation would require a multivariate analysis to optimally interpret several antibody scores without recourse to the binary categorisation. Such an approach might improve the herd-level test performance but might be unattractive to veterinary practitioners and to producers because it would involve complex and non-transparent calculations.

### The effect of herds characterised by intermediate within-herd antibody seroprevalence levels

The extent to which antibody seroprevalence can be used to determine herd-level status of infection is dependent on the level of separation of the frequency distribution of seroprevalence values in negative herds compared to the distribution in positive herds (see Fig. 1). The greater the separation of these frequency distributions, the easier it is to observe separate groups or classes of herds (aka “components” in the statistical literature) within the overall distribution. Various statistical methods exist for identifying and describing such classes or groups in a frequency distribution. The results we present here are predicated on the empirical description of such classes from a randomised study of Scottish beef herds prior to the Scottish eradication scheme^8^. The Scottish study found three classes, characterised by low, intermediate and high seroprevalence. Our analyses are based on the assumption that those groups represent different epidemiological herd statuses. The analyses are made complex by the fact that the epidemiological status of the group of herds characterised by intermediate levels of antibody seroprevalence is uncertain. Ideally we would have data from the same herds on both the within-herd seroprevalence for young stock and whether or not an infected animal exists in the herd. With such data we would be able to confidently describe the status of herds in the group with intermediate seroprevalence, but in the absence of such data we can consider the two extremes – i.e. that all herds in this group are either negative or positive. It is probable that this group of herds with intermediate seroprevalence consist of a mixture of truly positive and truly negative herds, in which case the correct interpretation would lie between the two extremes presented here.

It is worth considering the wider evidence regarding the existence of a group of herds with intermediate seroprevalence levels. Other studies have presented data suggesting the possible existence of such a group^11,12^. On the other hand at least one study exists in which there was no obvious middle component in the distribution of within-herd seroprevalence^5^.

There are various possible causes of intermediate seroprevalence in some herds:

- Existence of residual maternal antibody derived from dams in young stock that have not, in fact, been exposed to the virus.
- The young stock have been vaccinated.
- The young stock were exposed to low levels of virus (for example, over fence from a PI or transiently infected animal) leading to a low antibody response.
- Fomite contamination – leading to low antibody response and hence low prevalence.
- Exposure to a particular strain of virus that causes low antibody response.
- Heterogeneity in ages of the sampling group – if there are some new recruits to the group post-exposure they will be antibody negative and will “dilute” the group and hence reduce the prevalence.

In our analyses we have combined the intermediate seroprevalence group with either the low seroprevalence (negative) group or the high seroprevalence (positive) group. In doing so, we have effectively created a mixture of two, rather than three, groups. The resulting mixture depends upon the relative proportional contribution each group gives and we have, implicitly, used the relative proportions outlined in the original description of the three groups (Fig. 1). It is likely the contribution that the intermediate seroprevalence group makes to the overall distribution is not constant across time or between countries. It is not clear, however, whether we can predict how its contribution will vary. For example, in the case of a country or region with an eradication scheme such as that in Scotland, it is possible to envisage either an increase or decrease in the proportion of herds that fall into the intermediate seroprevalence category. During an eradication scheme, if all goes well, there is “movement” of herds from the right hand end of the seroprevalence frequency distribution (Fig. 1) towards the left hand end of the distribution. Therefore it is not possible to predict whether the middle group will gain more from the high seroprevalence group than it loses to the low seroprevalence group.

### Consequences of misunderstanding the status of the intermediate seroprevalence group

The large differences in herd-level test characteristics such as sensitivity, specificity, false negative and false positive rates, depending on whether the intermediate seroprevalence group is assumed to be negative or positive (Figs 2, 3 and 4; Tables 1 and 2) highlight the importance of better understanding this group’s epidemiological status if we wish to improve the accuracy of our estimates of these test characteristics. For example, in Fig. 2, it appears that, for a sample size of ten young stock, the trade-off between herd-level sensitivity and specificity is optimal with a cut-point of two if the middle component is positive but it is a cut-point of six or seven if the middle component is negative. Therefore the consequence of misunderstanding the status of the intermediate seroprevalence group is large – and in particular may lead to poor selection of the number of animals to test and poor selection of cut-points.

It is also worth noting that whatever the true status of intermediate seroprevalence group, the assumption that it represents truly positive herds results in estimates of the herd-level sensitivity which are lower than, or equal to, the actual herd-level sensitivity: this then represents a “worse case” estimate of herd-level sensitivity. It is therefore a “conservative” estimate for herd-level sensitivity. If this group were composed either partly or wholly of truly negative herds, then the actual herd-level sensitivity would be higher than predicted on the basis of the assumption that they are all truly positive. The opposite relationship can be seen for herd-level specificity: assuming that the intermediate seroprevalence group is positive results in estimates for this parameter which might be higher than the actual value. Whether it is preferable to under-estimate herd-level sensitivity or specificity of a test may be dependent on other conditions, as discussed below. Which of these test characteristics (sensitivity or specificity) is more important may determine which assumption, regarding the intermediate prevalence group, is the more “conservative” or risk-averse at a particular time.

### The final stages of an eradication scheme

We may also consider the relative importance of herd-level sensitivity and specificity towards the end of an eradication scheme when the prevalence is low compared to the early stages of an eradication scheme when the prevalence is high. In these latter stages of an eradication scheme it becomes more important that positive herds are accurately identified than it is in the early stages when it is typically accepted (and is less consequential) that some positive herds may be incorrectly classified as negative. This is because as the population approaches total susceptibility, the epidemiological, economic and political consequences of reintroduction become substantial^6^. Therefore it is important that the test sensitivity is particularly high during the final stages of eradication even if this comes with the increased risk of false positives. To achieve a meaningful and maximum sensitivity indicates a cut-point of just one antibody positive animal. Thus the overall probability of a false negative is reduced if the number of animals tested is increased to ten and the cut-point is held at one (Figs 2, 3). The consequent high risk of false positives due to the low specificity (Fig. 2 and Table 2) will require diplomatic explanations to farmers and veterinary practitioners. Specifically the explanation needed is that an initial positive result requires subsequent testing before the result is accepted as genuinely positive. Typically this subsequent test could be a full herd antigen screening for PI animals. Such a two-stage process in response to an initial positive result is a reasonable way of screening a herd before declaring its status. The proposed scheme in the Netherlands is based on follow up tests if the number of positive animals is equal to or higher than the cut-point (which is two out of five young stock in this example).

## Conclusions

In conclusion, we find that:

- The true status of the intermediate seroprevalence group is crucial, and analyses of herd-level sensitivity and specificity are highly dependent on the assumed status of this group. Misunderstanding the true status of this group will lead to imperfect design of a screening scheme and in particular poor selection of cut-points;
- Reducing the number of young stock screened for BVDv antibody from ten to five need not result in a substantial drop in herd-level sensitivity and specificity if the appropriate cut-point is selected;
- Increasing the cut-point to a value greater than one for the number of antibody seropositive animals before treating a herd as positive may be useful at the outset of an eradication scheme; The benefit of doing so is to increase the specificity, and reduce the cost of unnecessary secondary whole herd screening that is associated with a false positive.
- Increasing the number of animals tested, reducing the cut-point to one, or adopting both actions in the latter stages of an eradication scheme is warranted in order to maximise the herd-level sensitivity and thus reduce the number of false negative herds to as great an extent possible.

We believe this paper provides a useful example of a framework for understanding the complexity of measuring herd exposure to an infectious agent using a spot-test approach. It enables us to consider how the ‘test’ should evolve over the course of an eradication scheme.

## Methods

We estimated the herd-level sensitivity and specificity of a spot test of young stock using the frequency distribution of the number of BVD antibody-positive animals within a sample of young stock from Scottish beef suckler herds. Our starting point was the modelled distribution of a mixture of components for the number of positive ELISA antibody tests out of ten as published by Brülisauer *et al*.^8^ identified and described three statistically distinct groups or classes of herds on the basis of their within-herd seroprevalence levels: these groups are characterised by low, intermediate and high antibody seroprevalences. Brülisauer *et al*.^8^ described these groups statistically thus making them amenable to simulation (Fig. 1).

We hypothesised that each group reflects an epidemiologically important class of herd with the group characterised by low seroprevalences reflecting negative status herds and the group with high seroprevalences reflecting positive status herds. The status of herds with intermediate seroprevalences remains unclear and has several possible explanations^8,11,12^ (see Discussion above). We explore here the consequences of both the extreme scenarios that this intermediate group is fully negative or fully positive whilst recognising that it could be a mixture of both negative and positive herds.

We then envisaged the relatively high herd-level prevalence which might be expected at the outset of an eradication scheme and compared it with the relatively low prevalence anticipated in the latter stages. We sought to demonstrate the varying rate of false negatives, false positives and predictive values dependent on the prevalence as well as the screening design.

**Figure 6:**
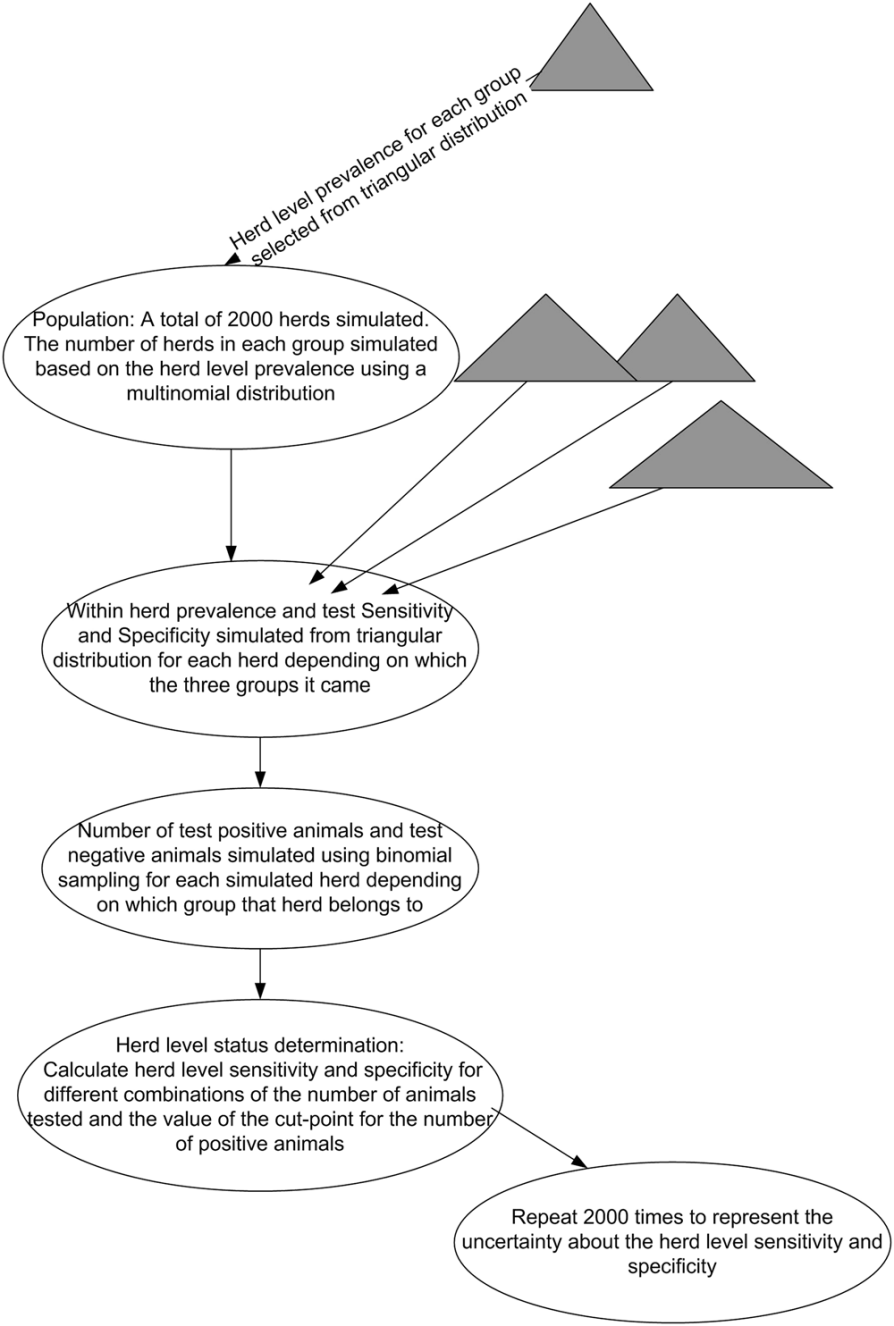
Flow diagram representing the process by which herd-level sensitivity and specificity (and uncertainty around these) was generated using the published estimates for animal-level antibody seroprevalence and test performance for each of the components identified in the source paper^8^.

The simulation was carried out using the following procedure which is also illustrated in a flow diagram (Fig. 6).

### The simulated population

We constructed a population of 2000 simulated herds (we chose 2000 because this is the approximate number of suckler herds in the sampling frame of Scottish population originally studied), which were distributed among the three classes of herds described above (i.e., herd classes characterised by low, intermediate, and high antibody seroprevalence levels within groups of young stock). The proportion of herds in each class was simulated based on published data^8^. We then simulated the true antibody seroprevalence in young stock within each herd (i.e., the overall proportion of young animals in each herd that are truly antibody positive), based on the range of seroprevalence values reported for the class of each herd. All simulation parameter values were based on published estimates, as presented in Table 3.

**Table 3:** The parameters and their 95% credible intervals from^8^, and consequent triangular distributions^25^used to simulate the number of true positives and the number of test positives (see Supplementary Information for more detail).

Parameter	Published 95% Credible Interval	Triangular distribution (min, mode, max)
*π*_1_ The proportion of herds characterised by low within-herd seroprevalence	(0.623, 0.742)	0.606, 0.683, 0.759
*π*_2_ The proportion of herds characterised by intermediate within-herd seroprevalence	(0.113, 0.213)	0.099, 0.163, 0.227
*π*_3_ The proportion of herds characterised by high within-herd seroprevalence	(0.116, 0.197)	0.104, 0.157, 0.209
*µ*_1_ The within-herd seroprevalence for herds with low seroprevalence	(0,0)	NA
*µ*_2_ The within-herd seroprevalence for herds with intermediate seroprevalence	(0.263, 0.385)	0.245, 0.324, 0.403
*µ*_3_ The within-herd seroprevalence for herds with high seroprevalence	(0.919, 0.998)	0.908, 0.959, 1.000
*S*_*e*_ The sensitivity of the individual (animal-level) test for BVDv antibody	(0.919, 0.998)	0.908, 0.959, 1.000
*S*_*p*_ The specificity of the individual (animal-level) test for BVDv antibody	(0.980, 0.993)	0.978, 0.987, 0.995

### Testing for antibodies to BVDv

From each simulated herd, a sample of a fixed number of animals was drawn (the exact sample size varied depending on the scenario: see below for more detail). No assumption for the total number of young stock in the herd was made as this was not necessary – it was merely assumed that each herd had sufficient young stock to fulfil the desired sample. The true status (antibody-positive or antibody-negative) of each animal in the sample was determined based on a random binomial distribution defined by the overall proportion of antibody-positive animals in the herd. The test status of each animal in the sample was then determined on the basis of its true status and the (imperfect) test performance parameters (test sensitivity and specificity at the individual-animal level) drawn from the triangular distributions described in Table 3.

### Herd-level status determination

We then envisaged a cut-point for the number of test-positive animals in the sample at, or above which, the sampled herd would be deemed to be positive. If the number of test positives was below that cut-point then the herd was interpreted as being negative.

### Calculating herd-level sensitivity and specificity

Therefore, we had 2000 herds each of which was from one of the three classes of herd (characterised by low, intermediate or high antibody seroprevalence). The low-seroprevalence class of herd was described as negative, and the high-seroprevalence as positive. The intermediate seroprevalence group of herd was treated as either negative or positive depending on which scenario we selected. This status was, in effect, our (simulated) “true” status for the herd. For each herd we also had the number of individual test-positive animals, and therefore when compared to a chosen cut-point the herd was designated “test positive” or “test negative”. As the test is not perfect, some herds may be truly negative but test-positive and vice versa. By summing over all herds we then calculated the proportion of “true” positive herds that tested positive and similarly for negative herds to estimate the herd-level sensitivity and specificity of the screening regimen.

### Herd-level specificity and sensitivity under different scenarios

Different scenarios were examined to explore the consequences of important contributing factors on the predicted herd-level specificity and sensitivity:

- Different sample sizes (the number of young stock tested);
- Different cut-points; and
- Different assumptions about the group of herds characterised by intermediate seroprevalence.

### False negative, false positive rates and predictive values

The resulting simulated herd-level sensitivity and specificity estimates were used to estimate rates of false negatives and false positives and to estimate positive and negative predictive values under two different scenarios: (a) high BVDv prevalence as typical of the early stages for an eradication scheme and (b) low BVDv prevalence as typical of an “end-game” scenario towards the latter stages of an eradication scheme. The high BVDv herd-level prevalence was set at 60%^8,21–23^. The low BVDv herd-level prevalence scenario was hypothesised at 0.5% based on work by Løken and Nyberg (2013)^24^ in which after 7 years of eradication the prevalence had dropped to 0.5%.

### Simulating uncertainty

Each run of the model provided a single estimate of the herd-level sensitivity and specificity, based on 2000 herds. In order to estimate the *variation* or uncertainty, we ran the model 2000 times (to provide a 95% precision of ± 0.5% on a proportion of 1%), thus giving a range of values for herd-level sensitivity and specificity.

### Data availability

The data used in this study were all generated through simulation based on the statistical distributions described in a publicly available paper^8^.

## Electronic supplementary material

Supplementary Information
