# Supplementary material for: Strategies for screening young stock for antibodies – optimising numbers to test, cut-points, & predictive values for bovine viral diarrhoea virus
Source: Sci Rep. 2018 Jun 22;8:9532. doi: 10.1038/s41598-018-27870-8 (PMC6014978; doi:10.1038/s41598-018-27870-8)
Supplement: Supplementary file 1 — Supplementary Information [file 41598_2018_27870_MOESM1_ESM.pdf]

Supplementary information

Strategies for screening young stock for antibodies – optimising numbers to test, cut-points, & predictive values for bovine viral diarrhoea virus.

Humphry*, R.W., Reeves, A., & Gunn G.J.

Epidemiology Research Unit, (Inverness campus), Scotland's Rural College (SRUC), Kings Buildings, West Mains Road, Edinburgh, EH9 3JG, U.K.

*Corresponding author. +44 1463 246059

E-mail address: roger.humphry@sruc.ac.uk (R. W. Humphry)

Details of the random selection of parameter values from a triangular distribution based on 95% confidence interval (Figure S1).

Step 1: estimate the mode, the minimum and maximum for a triangular distribution based on a 95% confidence interval.

Let L1 and H1 be the lower and higher 95% confidence intervals.

We assume a symmetrical triangle and hence the mode, M, is assumed to be the mid-point between L1 and H1 and thus easily calculated M=(L+H)/2.

The minimum *a* of the triangle is then found:

Area of left hand half triangle

$A = 0.5 = (M - a) * g$ *Equation 1*

Area of small triangle in left hand corner = ½ * (1-0.95) = 0.025 (because *L* represents the lower 95% confidence limit).

$0.025 = (L - a) * h$ *Equation 2*

These two triangles are similar and therefore

$\frac{g}{h} = \frac{M-a}{L-a}$ *Equation 3*

Rearranging Equation 3: $g = h * \frac{M-a}{L-a}$ *Equation 4*

Substitution of Equation 4 into Equation 1:$0.5 = (M - a) * h * \frac{M-a}{L-a}$

$=> h * (M - a)^2 = 0.5 * (L - a)$ *Equation 5*

Rearrange equation 2

$h = \frac{0.025}{L-a}$ *Equation 6*

Substitute for h in Equation 6 into Equation 5

$$0.025 * \frac{(M - a)^2}{L - a} = 0.5 * (L - a)$$

$$=> \ 0.025 * (M - a)^2 = 0.5 * (L - a)^2$$

$$\left\{\frac{L - a}{M - a}\right\}^2 = 0.05$$

$$=> L - a = (M - a) * \sqrt{0.05}$$

$$=> L - M * \sqrt{0.05} = a\left(1 - \sqrt{0.05}\right)$$

$$=> a = \frac{L - M * \sqrt{0.05}}{1 - \sqrt{0.05}}$$

This establishes *a*, the minimum of the triangular distribution but a check is necessary to ensure that it doesn't go below zero. The maximum of *a* and zero is used for the minimum of the triangular distribution.

Similarly maximum, b, of the triangular distribution is assumed to the lower of 1 and a+2*(M-a)

From the consequent triangular distribution random samples are then simulated.

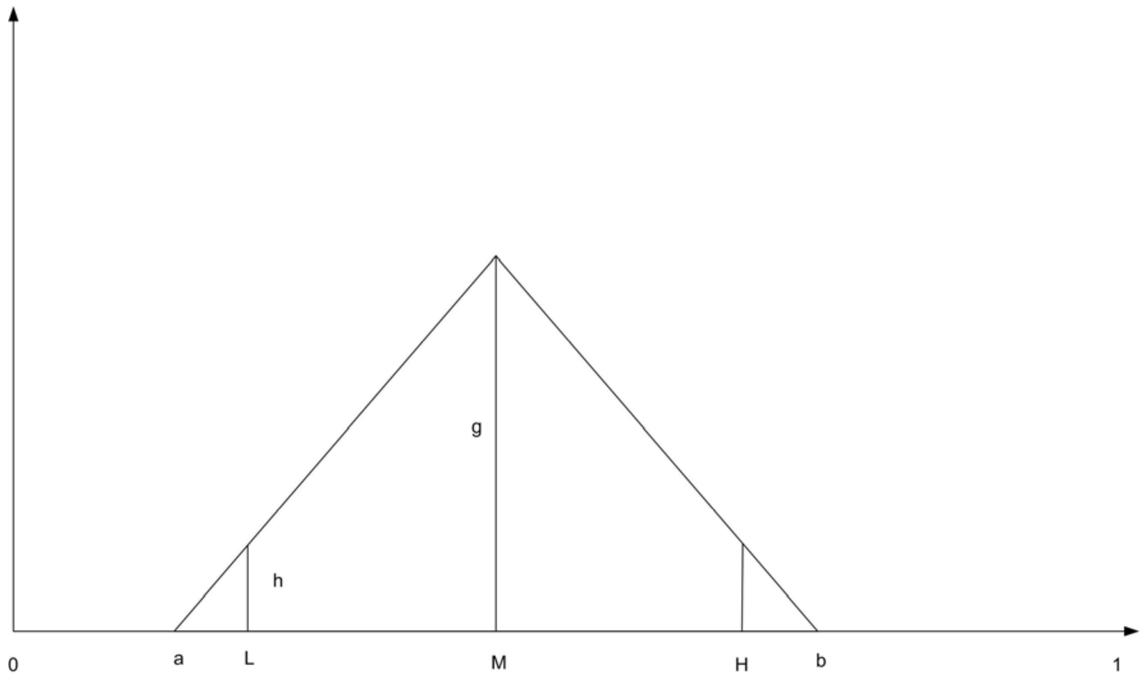

Figure S1, Supplementary Information. Diagram of the triangular distribution presenting the symbols used below to derive the parameters which describe the distribution for the purposes of simulating the uncertainty in the epidemiological parameter estimates such as sensitivity and specificity.
